# Novel synthesis of fibronectin derived photoluminescent carbon dots for bioimaging applications†

**DOI:** 10.1039/d2ra05137k

**Published:** 2022-10-26

**Authors:** Sara Strickland, Mychele Jorns, Lindsey Heyd, Dimitri Pappas

**Affiliations:** Department of Chemistry and Biochemistry, Texas Tech University Lubbock TX USA d.pappas@ttu.edu

## Abstract

Fibronectin (FN) derived from human plasma has been used for the first time as the carbon precursor in the top-down, microwave-assisted hydrothermal synthesis of nitrogen doped carbon dots (CDs). FN is a large glycoprotein primarily known for its roles in cell adhesion and cell growth. Due to these properties FN can be over expressed in the extracellular matrix (ECM) of some cancers allowing FN to be used as an indicator for the detection of cancerous cells over non-cancerous cells. These FN derived CDs display violet photoluminescence with UV excitation and appear to possess similar functional groups on their surface to their carbon precursor (–COOH and –NH_2_). This is believed to be due to the self-passivation of the CDs' nitrogen-containing surface functional groups during the heating process. These CDs were then used to stain MCF-7 and MDA-231 breast cancer cells and were observed to interact primarily with the cell membrane rather than intercalating into the cell like the many other types of CDs. This led to the hypothesis that the CDs are selectively binding to the FN overexpressed within the cancer cells' ECM *via* amide linkages. This is in agreement with the EDX and FTIR spectra of the FN CDs which indicate the presence of –COOH and nitrogen containing surface groups like –NH_3_. The inherent selectivity of the CDs combined with their ability to photoluminesce enables their use as a fluorophore for bioimaging applications.

## Abstract 2

Fibronectin (FN) derived from human plasma has been used for the first time as the carbon precursor in the top-down, microwave-assisted hydrothermal synthesis of nitrogen doped carbon dots (CDs).

## Graphical abstract

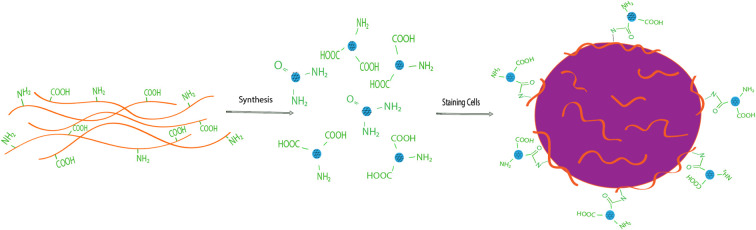

## Introduction

Carbon dots (CDs), carbon-based nanoparticles of 20 nm or less size,^1^ are widely applicable in a variety of fields due to their characteristic properties including high biocompatibility, simple post modification of surface groups, the ability to directly modify the produced particles by altering aspects of their synthesis method, and tunable photoluminescence/fluorescence properties.^2–4^ Nanoparticles are well known to have large surface areas compared to bulk materials, which in turn affect the binding efficiencies and photoluminescence properties.^5,6^ Furthermore, conjugating various bio-moieties to nanoparticles can be conducted to enhance their unique properties by enabling desired selectivity.^7^ However, the conjugation process can be tedious, expensive, and can leave the nanoparticle-bio moiety complex susceptible to degradation.^8–10^

CDs can potentially possess an inherent affinity for a type of cell or chemical, thus allowing the CD to be utilized as a biological sensor.^11–14^ This specificity combined with their ability to luminesce/fluoresce make CDs an ideal fluorophore for bioimaging applications. CD synthesis is relatively facile, requiring only a carbon source, a heating method, and purification of the sample of interest. These aspects can be varied to tune CDs properties to create the desired version of nanoparticle.^2^ In this study, to the authors' knowledge, photoluminescent CDs were synthesized for the first time using purified human plasma fibronectin (FN) as a carbon source.

FN is a large (*M*_W_ = 440 kDa) glycoprotein found in the extracellular matrix (ECM) of a wide variety of cells. FN is directly involved in the regulation of cell adhesion, multiplication, and mobility *via* integrin-mediated signaling.^15–17^ For cancer cells to proliferate, the physiological microenvironment must promote interaction between the normal cells in the nearby tissue and the cancerous ones. The mutation of an otherwise healthy cell into a cancerous cell involves composition changes of the cell's ECM, and it is these structural alterations to the ECM which can cause changes in cell behavior.^18–20^

Certain ECM glycoproteins then can be upregulated during these mutations including various collagens, biglycans and FN.^18,20,21^ Additionally, it has been reported that the presence of FN in cancer cells has been associated with cancer growth and subsequent metastasis. This is believed to be due to FNs role in stimulating cell growth *via* integrin-mediated intracellular signal transduction and its adhesive properties from a high number of specific binding sites for ECM proteins and cellular receptors like α5β1 integrin.^22–25^

In this study, a “top down” CD synthesis process^2,4^ was utilized. Human plasma-derived FN was used as the carbon source, then doped with nitrogen from the solvent (formamide) to increase the nanoparticles' photoluminescent properties^26–28^ and carbonized in a hydrothermal vessel using microwave radiation. Microwave heating was used due to its rapid formation of CDs with relatively homogenous size distribution and does not require surface passivation to protect the functional groups as the CDs grow.^29^

This study will also discuss the potential applications of these CDs as nontoxic fluorophores used in the study of extracellular matrix. Fluorescence microscopy was utilized to visualize the location of CDs in the cell, with subsequent characterization techniques to elucidate the chemical and physical properties of the nanoparticles. These properties can then be used to determine feasible applicability of these luminescent CDs for biological imaging. It is hypothesized in this study that these FN-derived CDs possess a selectivity for FN, which is overexpressed in the ECM of some adherent cancer cell lines. This inherent selectivity could likely be due to the synthesis process which allows for the CDs to maintain some of the carboxylic acid functional groups as surface groups from the original carbon source (FN) and therefore promotes binding to the primary amine also found in FN.^2–7^ Given this, these CDs can potentially be used as a sensor for FN or for the imagine of cell/tissue growth.

## Experimental

### Materials

Breast cancer cell lines (MCF-7 and MDA-231) were procured from the American Type Culture Collection (ATCC), cultured in RPMI 1640 medium (Hyclone), and incubated at 37 °C at 5% CO_2_. Fibronectin derived from human plasma was obtained from Corning. Ethylenediamine and formamide were obtained from Fisher Scientific. Acetone was obtained from Macron Fine Chemicals. Phosphate-buffered saline (PBS) was obtained from Mediatech, Inc. The sodium chloride (NaCl) was obtained from Sigma. The propidium iodide (PI) was obtained from Molecular Probes Invitrogen detection technologies. Poly-d-Lysine Cellware 35 mm Coverslip-Bottom Dishes obtained from Corning.

### General synthesis procedure for microwave FN

CDs 6.5 mg of FN was dissolved in 20 mL of formamide and vortexed until the carbon source was completely dissolved. Then 0.525 mL of ethylenediamine was added into the solution and vortexed until homogenous. The solution was then transported into the Teflon cup of the hydrothermal vessel (Parr Microwave Acid Digestion Vessel 4781). Due to the limited volume of this vessel the solution was split in half with each half and only half the solution was heated at a time. The vessel was then heated in the microwave (Panasonic NE-1054F, 1000 W output) at the medium–high power setting (700 W) for 20 seconds at a time. Each heating period was followed by a 30 minutes cool down period. This is repeated until a total of 160 seconds in the microwave is reached (8 rounds of 20 seconds of heating per vessel).

After the final cool down, the CD solutions were combined by filtering with a 0.2 μm pore Nylon membrane disk filter into a single container. The CDs were then precipitated out of the solution with 50 mL of acetone. The resulting solution was then transferred to Teflon centrifuge tubes (Thermo Scientific Oak Ridge FEP 50 mL) to centrifuge at 10 000 RPM in order to remove the supernatant and obtain the CD precipitate. The pellet was then washed twice using a 1 : 1 (v/v) acetone and ethanol mixture. Once washed, the CDs were transferred to a Petri dish so the wash solution can evaporate off leaving the dried CDs residue. Once this was completed the CDs could be suspended in 50 mL of DI water or left as a solid.

### Characterization of FN CDs

Ultraviolet-visible (UV-vis) absorption data was obtained using an Agilent 8453 UV-Vis Spectrometer. CD fluorescence spectra were obtained using the Agilent Cary Eclipse Fluorescence Spectrometer. Lifetime data was obtained using Horiba Johbin Yvon Single Photon Counting Controller Fluorohub with a MicroHR Horiba Jobin Yvon spectrometer. The lifetime decay of the FN CDs was determined using the equation:1
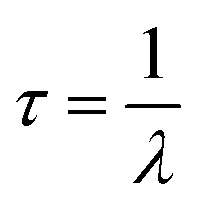
where *τ* is the lifetime of the CDs and *λ* is the decay constant.

Zeta potential obtained using Microtrac Stabino particle charge mapping system. Transmission electron microscopy (TEM) was conducted using the Hitachi H-9500 TEM rigged with an EDAX Energy dispersive X-ray (EDX) spectrometer. Thermo Scientific Nicolet iS10 Smart ITR FTIR with ATR crystal was used to obtain the FTIR spectrum.

### Biocompatibility experiment of CDs and cancer cells

5 vials holding 1 mL each of MCF-7 cells suspended in medium was obtained. These vials were dedicated for 1 control of unstained cells, 1 viability control of cells with PI, and then 3 replicates of cells incubated in the CDs solution and 10 μL propidium iodide. CDs solutions consisted of CDs resuspended in DI water, the CDs source vial was sonicated for about 5 minutes and then the volume required for the 3 replicate vials was removed and placed into a different vial. 30 μL of NaCl solution (8 g NaCl per 100 mL ultrapure water) was mixed into this CD vial to prevent unnecessary cell death from the addition of a hypotonic solution. Once the CDs staining solution was prepared, the 5 vials of cells were then centrifuged at 4500 RPM for 5 minutes. The medium was removed, and the cell pellet was resuspended in approximately 1 mL of PBS for the 2 control samples, and 1 mL of the CD and salt solution for the 3 replicate test samples.

The vials were then incubated for 45 minutes. At this point, 10 μL of PI was added to each vial except the unstained control vial then incubated for an additional 15 minutes. After incubation the vials were all centrifuged again under identical parameters, with all vials washed and resuspended in PBS three times. The resuspended vials were then transferred into flow cytometry tubes and analyzed with a Becton-Dickinson FACSCalibur flow cytometer to determine cell viability. PI is a membrane-impermeant dye that fluoresces with very high intensity when bound to cell DNA in the nucleus. This experiment was then repeated for the MDA-231 cell line.

### Bioimaging of cancer cells using FN CDs

2 vials of MCF-7 or MDA-231 cells (approximately 1 mL each) suspended in medium were obtained and then centrifuged at 4500 RPM for 5 minutes to isolate the pellet and remove the medium. Each pellet was then resuspended in approximately 1 mL of fresh medium and transferred to a coverslip-bottom dish with 1–2 mL of medium already in the dish. The cells were then incubated for 48 hours to ensure adequate growth. After this incubation period was completed the desired volume of CDs solution (with added concentrated NaCl salt solution to prevent lysing the cells as described in previous section) was added to 1 dish and the other dish was left untouched to serve as a control for cellular autofluorescence. The cells with the CDs were then incubated for an additional 40 minutes. Then the medium was removed from each dish and replaced with PBS. The cells were then imaged using a Nikon Eclipse Ti2-A inverted microscope with a Lumencor light engine as the excitation source and a PCO edge sCMOS camera. The cells were imaged using 20× and 60× objective lenses under white light and a UV light band-pass filter cube with an excitation wavelength center of 375 nm and bandpass of 28 nm, a excitation wavelength center of 460 nm and bandpass of 50 nm, and a dichroic mirror of 415 nm for fluorescence images. ImageJ software was used for image analysis.

## Results and discussion

**Fig. 1:**
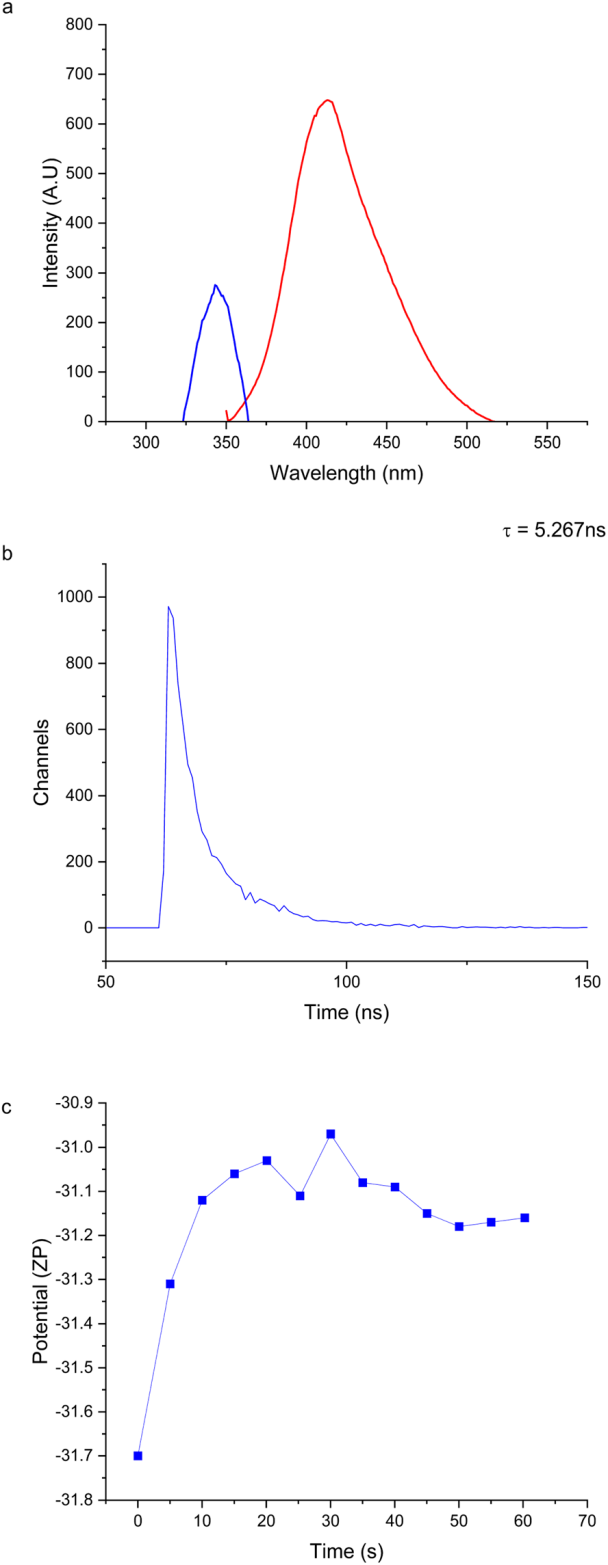
(a) Fluorescence excitation and emission spectra of 100 μL FN CDs suspended in DI water. The excitation spectrum (red) peak is 345 nm. The emission spectrum (blue) peak is 413 nm. (b) Photoluminescence decay lifetime of the FN derived CDs is 5.267 ns. (c) The zeta potential of the nanoparticles in DI water −31.15 ± 0.07 mV.

The synthesized FN CDs form a black precipitate that becomes murky grey/brown solution when sonicated. When irradiated with UV light the aqueous suspension of nanoparticles exhibits a blue/violet fluorescence. The fluorescence profile of these CDs was measured and found to achieve brightest fluorescence when excited under 345.07 nm UV light and emitting at 413.03 nm, indicating photoluminescence extending well into the UV and entering the visible spectrum (Fig. 1a).

These excitation and emission wavelengths are comparable to sulfur co-doped CDs that used l-cysteine and citric acid as the carbon precursors.^30^ The structure of FN is a dimeric glycoprotein with large subunits (220–250 kDa) linked *via* disulfide bonds.^15^ These disulfide bonds may still be present in the carbon precursor. These CDs were excited under 345 nm UV light to reach maximum emission wavelengths of 415 nm. This is believed to be due to the surface of the CD being doped with nitrogen and sulfur atoms causing electron/hole recombination. The sulfur heteroatoms increase the radiation transition of the nitrogen containing surface groups.^30^ This is due to the six valence electrons in the sulfur helping embed the nitrogen and the sulfur into the carbon skeleton, thus increasing the likelihood of electronic transitions occurring.^30,31^

**Fig. 2:**
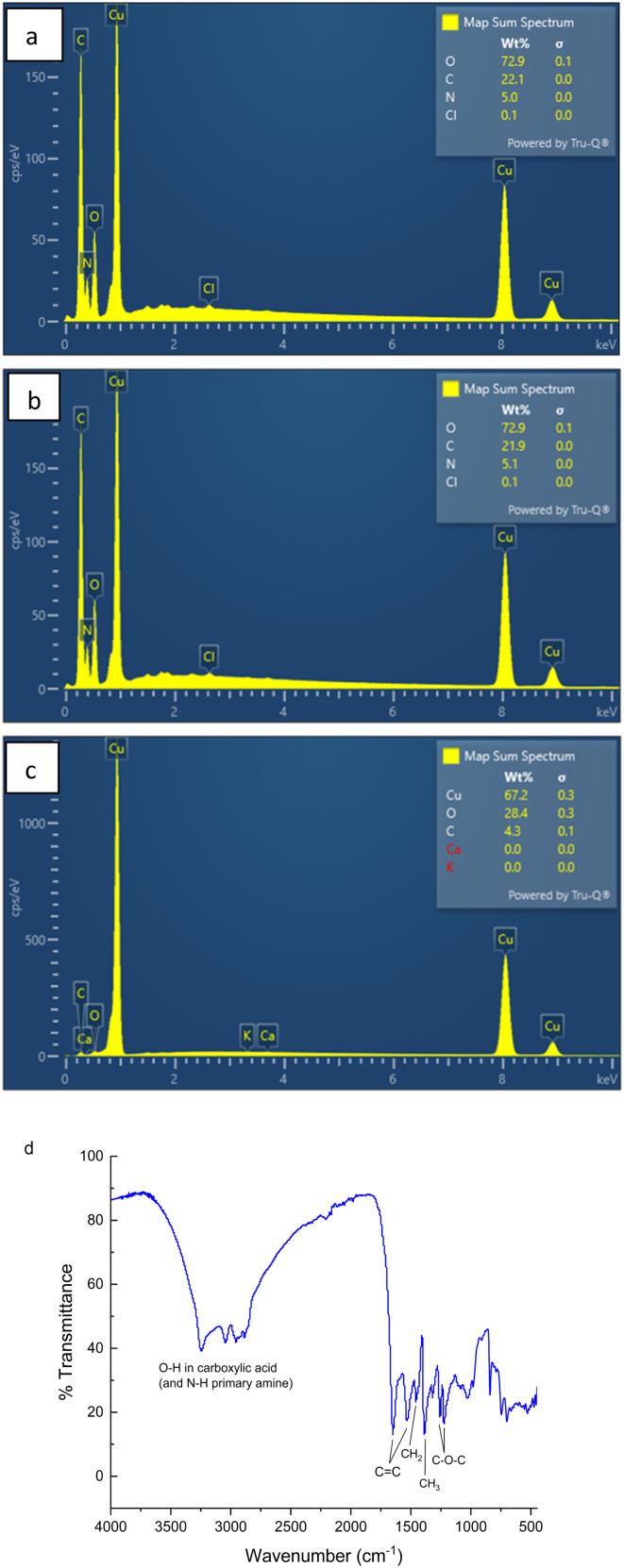
(a) EDX spectroscopy mapping of total elemental composition of FN derived CDs at locations 1–3. The sum weight percent of oxygen is 72.9%, the sum weight percent of carbon is 22.1%, the sum weight percent of nitrogen is 5% and the sum weight percent of chlorine is 0.1%. The large copper peak is due to the copper substrate utilized during analysis. (b) EDX spectroscopy mapping of total elemental composition of the CDs at locations 5 and 6. The sum weight percent of oxygen is 72.9%, the sum weight percent of carbon is 21.9%, the sum weight percent of nitrogen is 5.1% and the sum weight percent of chlorine is 0.1%. The large copper peak is due to the copper substrate utilized during analysis. (c) EDX spectroscopy mapping of overall elemental composition of locations 7–9 of the copper slide. The sum weight percent of copper is 67.25, the sum weight percent of oxygen is 28.4% and the sum weight percent of carbon is 4.3% (d) FTIR spectrum of FN derived CDs indicates the presence of O–H stretch in carboxylic acid groups, N–H stretch in primary amines, C=C double bonds, CH_2_ bends, CH_3_ bends and C–O–C stretches.

The photoluminescence emission seen in these CDs can also be attributed to other surface groups like –COOH and –OH. Nitrogen containing surface groups cause a red shift in the photoluminescence emission, while the more blue shifted emission CDs have a higher incidence of oxygen and carbon in the surface groups.^30^ It has been demonstrated by one research group that by using formamide as the primary solvent they were able to produce more blue emitting CDs even with nitrogen containing surface groups.^32^ Therefore, the violet emission of these FN CDs supports this idea that the functional groups are primarily composed of carbon and oxygen (Fig. 2). The photoluminescent properties of FN CDs may be due to the synergistic effect of co-doping sulfur and nitrogen.^30^

It is important to mention that the sulfur used in this synthesis comes entirely from the disulfide bonds and sulfur containing amino acids located in the peptide bonds of the FN protein.^15^ Overall, this is a very small amount of sulfur, especially when compared to the amount of carbon, oxygen and nitrogen being introduced into the system *via* the carbon precursor and solvent. The effect of doping of this small amount of sulfur is likely to only marginally increase the nitrogen's ability to implant itself into the carbon skeleton. This also explains why we do not see a major red shift in the CDs photoluminescence despite the presence of nitrogen-containing surface groups.

The photoluminescence decay lifetime was calculated to be 5.267 ns (Fig. 1c). This lifetime measurement is comparable to other CDs, particularly those doped with nitrogen, as it is believed that the presence of nitrogen in surface functional groups extends the lifetime decay of CDs.^30^ This is thought to be due to the increased number of –NH_2_ groups which act as electron donors. This assists recombination of electrons held at the surface in electro donating groups with electron holes in the CDs. This promotes electrons into higher energy states and increases fluorescence emission efficiency *via* self-pacification^27,30,32–37^

Elemental composition spectra of three different locations of the dried FN derived CD sample were analyzed (Fig. S2 and S3†). Fig. 2a–c represents the overall weight percent of each element. Chlorine and sodium are likely contaminants in sample handling. As indicated by the graph, the higher oxygen and carbon content are higher compared to the nitrogen content, in agreement with fluorescence measurements.

However, the presence of nitrogen indicates that the use of formamide and ethylenediamine to dope in the nitrogen was successful. We suspect that due to the small amounts of sulfur in the carbon precursor it was not picked up in the EDX spectrum. It was also noted that the elemental composition of the FN derived CDs was uniform throughout all the locations mapped (Fig. S2 and S3†). In Fig. 2c it is shown that the copper substrate included a substantial oxygen peak likely due to the formation of copper oxide on the substrate surface. This could skew the elemental mapping of the FN CDs and make the oxygen content seem artificially high.

The FN CD FTIR spectrum (Fig. 2d) has a broad group of bands from 2880 cm^−1^ through 3250 cm^−1^, which is characteristic of the O–H stretch of carboxylic acid and the N–H stretch of primary amines. This group of bands is made of peaks at 2880 cm^−1^, 2950 cm^−1^, 3050 cm^−1^ and 3250 cm^−1^. The 1650 cm^−1^ and 1530 cm^−1^ bands are associated with the C=C bond we believe comes from the core of the nanoparticle.^2,13,29–31^ The 1460 cm^−1^ band is associated with the CH_2_ bend and the 1390 cm^−1^ band is associated with the CH_3_ bend. Lastly, the 1260 cm^−1^ frequency indicates the C–O–C stretch.

**Fig. 3:**
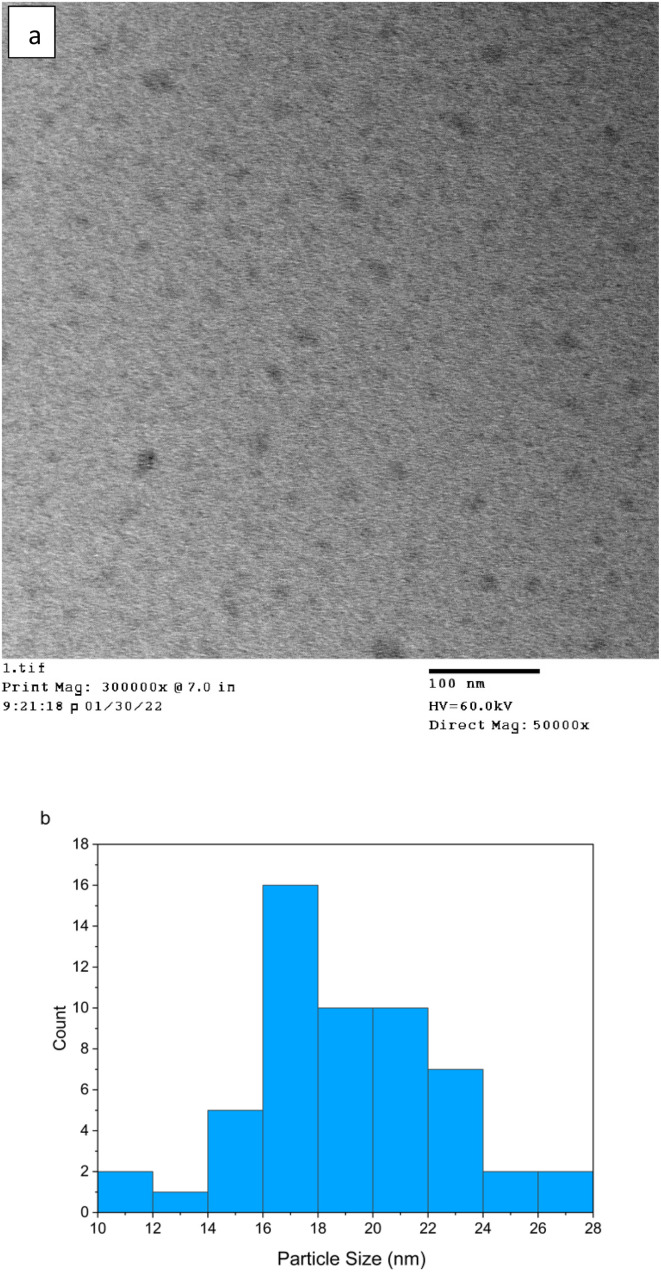
(a) TEM image of FN derived CDs indicating the presence of semi-spherical particles distributed throughout the image. (b) Size distribution of FN precursor CDs indicates the average size nanoparticle to be about 17 nm. The smallest nanoparticle measured is 10 nm and the largest is 28 nm.

In terms of physical characteristics of the FN CDs, the TEM images of Fig. 3a indicate the FN-derived CDs have varying sizes with the average size approximately 17 nm (Fig. 3b). These protein-derived CDs are larger than typically achieved with smaller precursors, which could be due to the increased overall microwave heating time.^1,29^ Due to this we hypothesize that the larger carbon precursor size leads to the formation of a larger C=C core.

**Fig. 4:**
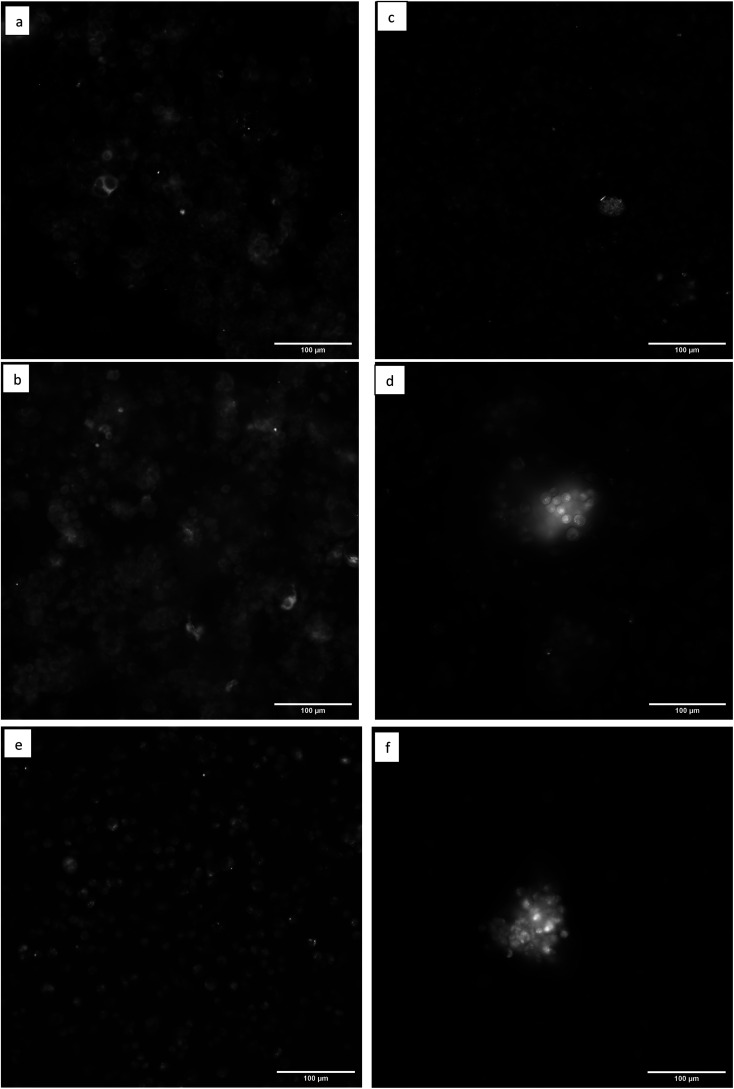
(a) MCF-7 cell line control under UV excitation shows clear difference in intensity of autofluorescence of cytosol and the edge of the cells membrane. (b) MCF-7 cell line stained with 100 μL of FN CDs under UV excitation indicates substantial decrease in contrast between cytosol and cell membrane, indicating presence of FN CDs across the cell surface. (c) MDA-231 cell line control under UV excitation indicating clear contrast of autofluorescence intensity between cytosol and the edge of the cells membrane. (d) MDA-231 cell stained with 80 μL of FN CDs indicates presence of FN derived CDs at cell membrane due to the decrease in autofluorescence contrast of the cytosol and cell membrane. (e and f) This image depicts MDA-231 cell line stained with 100 μL of FN CDs and 80 μL of FN CDs respectively. These images highlight the increased photoluminescence intensity seen with cancer cell clusters stained with FN CDs.

Fluorescence microscopy images of the MCF-7 and MDA-231 breast cancer cells in Fig. 4 reveal that both cell lines have distinguishable differences in photoluminescence patterns between the control and FN CD-stained cells. The autofluorescence in Fig. 4a shows an increase in fluorescence emission around the edges of the cell thought to be associated with the cellular membrane and ECM with the less intense emission in the center thought to be associated with the cytoplasm. However, it can be seen in Fig. 4b that this autofluorescence contrast is less distinct than when the FN CDs are binding to the surface of the cell. The tendency of the FN CDs to favor the cell surface instead of the cytoplasm is significant since most non-affinity CDs tend to naturally be interpolated into the cell.^2^ This surface binding is believed to be due to selective binding of the FN CDs to the cellular FN found in the ECM of the MCF-7 cells. This effect is found to be consistent throughout the cells in this image.

Fig. 4c is representative of the typical autofluorescence seen for the MDA-231 cell line. When the cells were stained with the increasing volume of FN CDs (Fig. 4d–f) the cells showed similar qualitative traits as the MCF-7 cells, with the distinct outline of the cell originating from autofluorescence in the cell membrane being less defined. This further demonstrates that the CDs are selectively binding to the surface of the cell, regardless of cell line, instead of passively diffusing through the cell membrane or congregating into an organelle. It is also seen that when CD-stained cancer cells cluster together the photoluminescence increases greatly. This also coincides with our proposed binding theory since the FN in the ECM primary functions in cellular adhesion and multiplication and is thought to promote increased cancer growth.^4–7^ Ergo, if there is a cluster of cancer cells, their ECM is more likely to be flush with FN protein and there will be a higher level of specific binding of FN selective CDs.

**Fig. 5:**
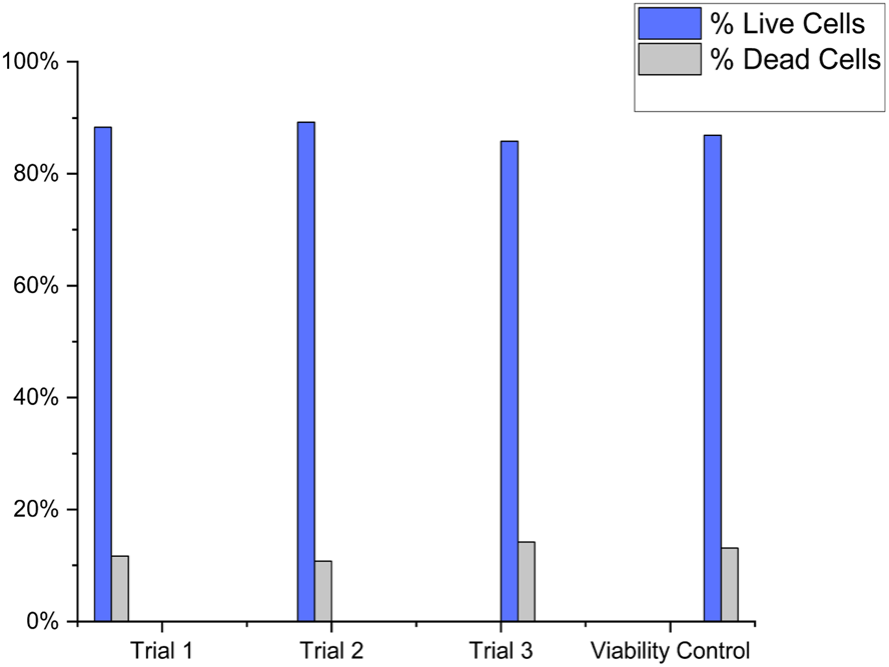
This image depicts the biocompatibility of cells stained with 100 μL of FN CDs. The percentage of cell death for cells stained with 100 μL Fn CD was determined to be 11.7%, 10.8%, and 14.2% respectively. Percentage of cell death for viability control was determined to be 13.1%. The percentage of live cells in viability control was found to be no statistical difference between viability control and cells stained with the FN CDs.

To measure the biocompatibility of the FN derived CDs with cells, the MDA-231 cell line was incubated with CDs and tested for membrane exclusion staining. The viability control of the biocompatibility experiment was the MDA-231 cell line stained only with PI (Fig. 5). Three samples of MDA-231 cells were stained in an identical manner with PI and 100 μLs of FN CDs for three triplicate runs. The percentage of dead cells in Fig. 5a was determined to be 13.1%. The percentage of dead cells (Fig. 5b–d) was found to be 11.7%, 10.8% and 14.2% respectively. The average percentage of cell death when stained with FN derived CDs was 12.2%. A *t*-test was performed to determine if the percentage of dead cells from the viability control were statistically different from the FN CD-stained cells. At a 95% CI the two-tailed *p* value equals 0.4715, thus the viability control and FN CD-stained cells are not considered statistically significant. Therefore, these CDs are not toxic to cells and could potentially be used *in vivo*.

## Conclusions

Photoluminescent CDs were formed using fibronectin protein as the carbon precursor. These CDs while hydrophilic and small enough to enter the cell^2^ (∼17 nm), the FN derived CDs appear to interact primarily with the ECM of MCF-7 and MDA-231 breast cancer cell lines. The selectivity of these CDs is believed to be due to the functional groups present in Fig. 3d FN, –COOH and –NH_3_.^15,22^ Regarding the nitrogen-containing surface groups, the hypothesis is that these groups cause the surface of the CD to become passive, and the self-passivation during synthesis preserves these functional groups originating from the carbon precursor which facilitates selective binding of the CD to FN.^27,29,30,32–37^ In addition, by implementing MW radiation with hydrothermal vessels, the nanoparticles are able to form without major impact of the surface groups due to the self-passivated layer.^29^ These FN selective CDs could have potential in the bioimaging field as FN has been known to be over-expressed in the ECM of certain cancer cell lines believed primarily to be due to its roles regulating cell adhesion and growth.^15,18,22,23^ The photoluminescent ability of these FN derived CDs paired with FN selectivity allows for the specific tagging of cancer without the need to conjugate the photoluminescent nanoparticle to an antibody or some other bio moiety. This would negate the issues other nanoparticle-bio moiety complexes have like increased instability leading to complex degradation, altering of the fluorescent ability of the fluorophore/nanoparticle or biocompatibility issues.^8^ Ultimately, the ability of FN CDs to photoluminesce while maintaining biocompatibility and selectively binding to FN makes these nanoparticles suitable for bioimaging applications.

## Author contributions

The authors would like to acknowledge Dr Kristen Hutchins and Mr Gary George III for access to FTIR instrumentation, and Dr Bo Zhao in the TTU Imaging center for TEM and SEM-EDX analysis.

## Conflicts of interest

There are no conflicts to declare.

## Supplementary Material

RA-012-D2RA05137K-s001
