# Supplementary material for: Novel synthesis of fibronectin derived photoluminescent carbon dots for bioimaging applications
Source: RSC Adv. 2022 Oct 26;12(47):30487–94. doi: 10.1039/d2ra05137k (PMC9597609; doi:10.1039/d2ra05137k)
Supplement: RA-012-D2RA05137K-s001 [file RA-012-D2RA05137K-s001.pdf]

Electronic Supplementary Material (ESI) for RSC Advances.
This journal is © The Royal Society of Chemistry 2022

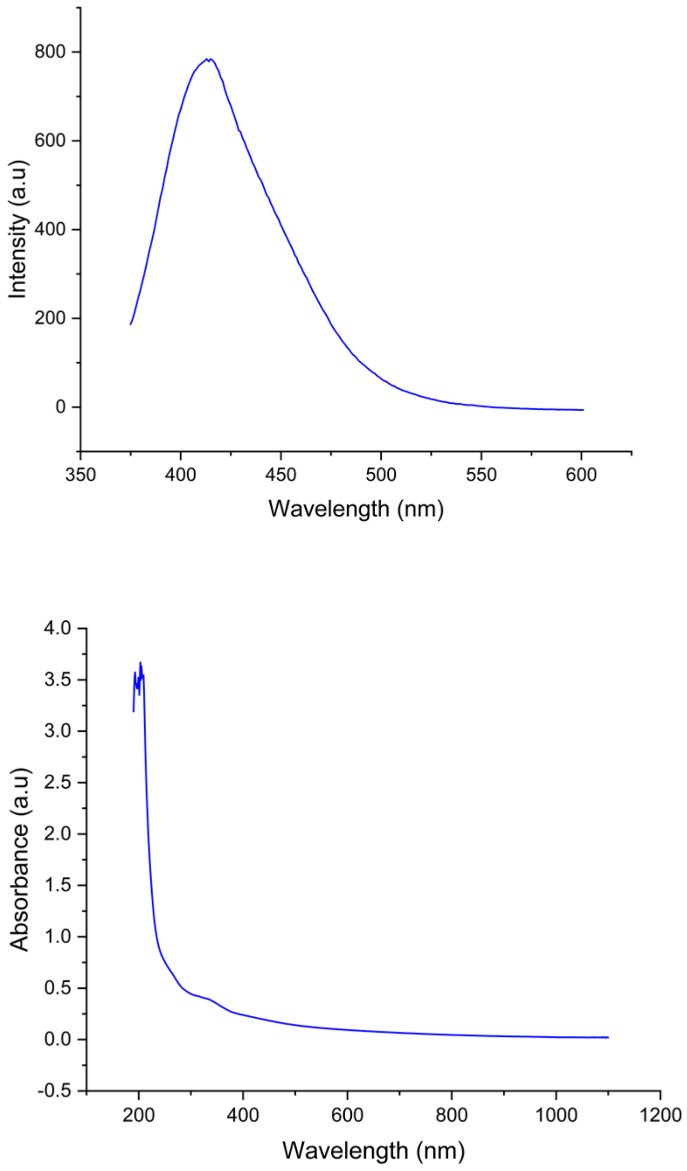

Supplementary Figure 1: (a) Emission spectrum of FN CD at 10% concentration under 345 nm excitation. The integrated intensity of this spectrum used to determine QY. (b) Absorbance spectrum of concentration FN CD at 10% concentration, value at 345 nm used to determine QY.

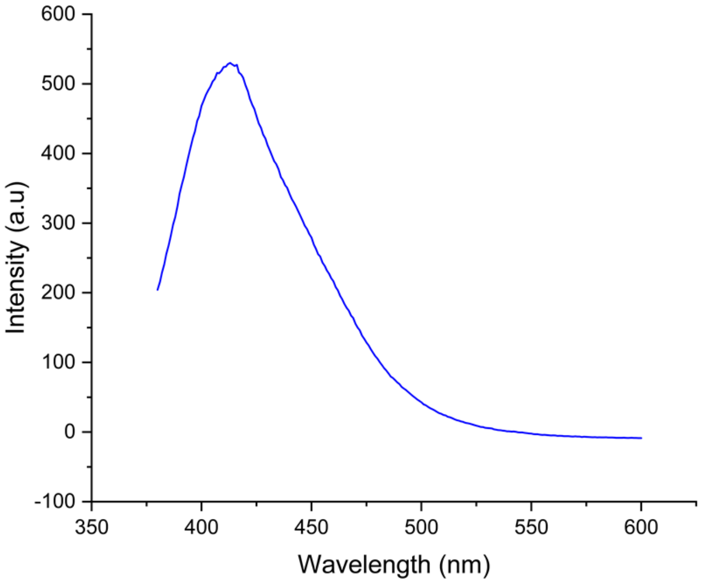

Supplementary Figure 2: Emission spectrum of FN CD at 5% concentration under 345 nm excitation, 8 months later.

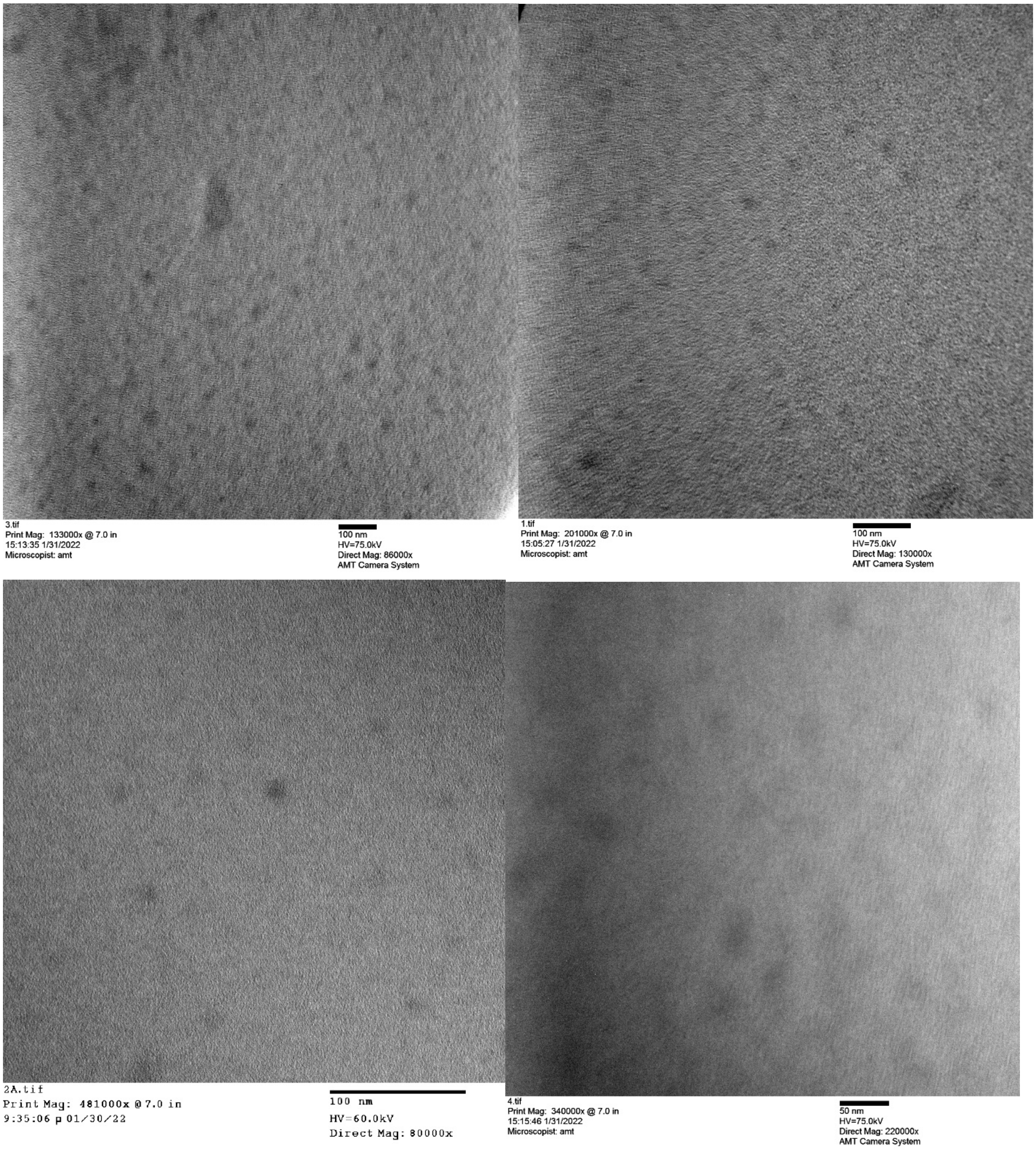

Supplementary Figure 3. (a) TEM image of FN derived CDs at direct magnification 86,000x. (b) TEM image of FN derived CDs at direct magnification 130,000x. (c) TEM image of FN derived CDs at direct magnification 8,000x. (d) TEM image of FN derived CDs at direct magnification 220,000x.

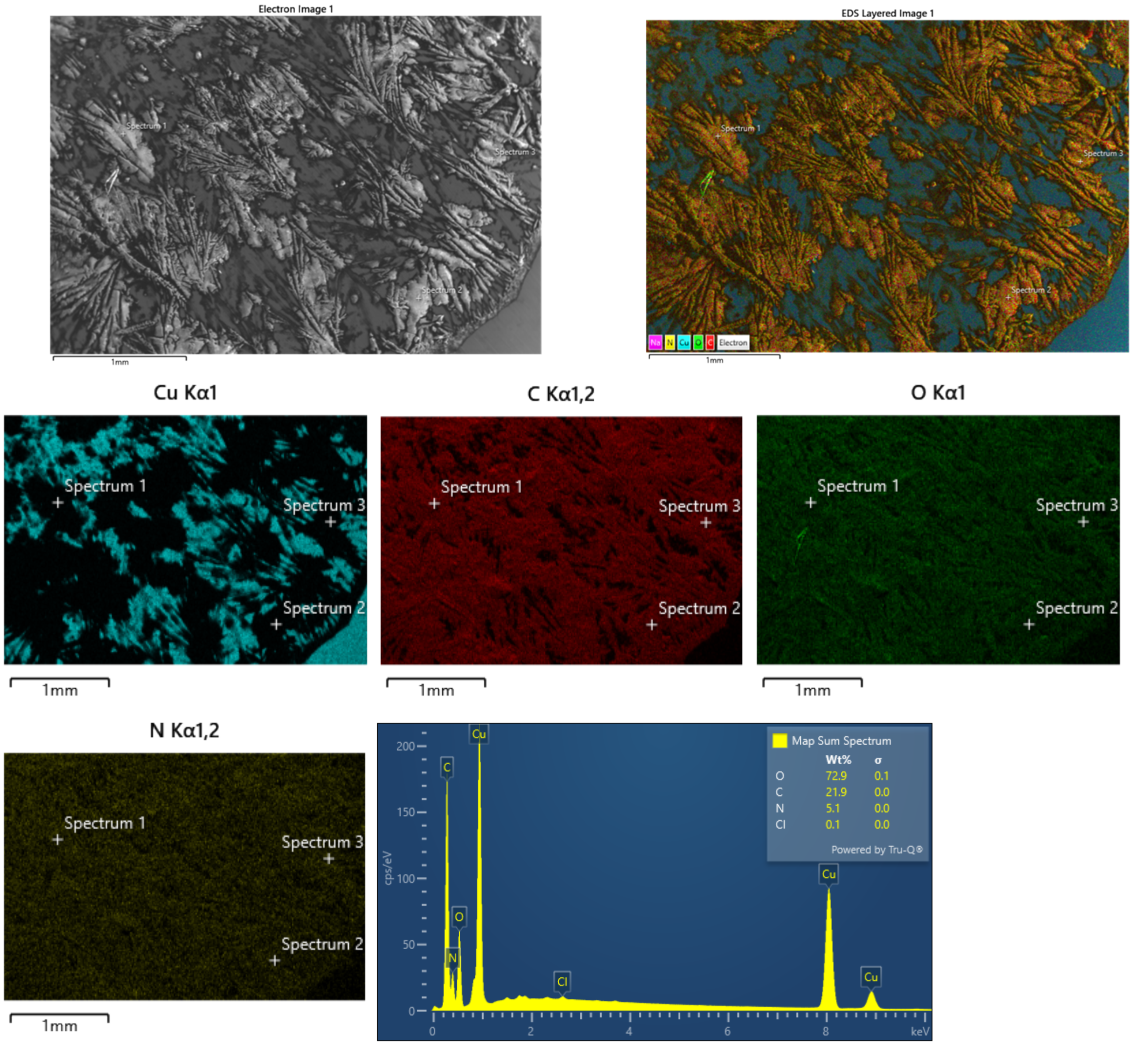
Electron Image 1
EDS Layered Image 1
Cu Kα1
C Kα1,2
O Kα1
N Kα1,2
Spectrum 1
Spectrum 2
Spectrum 3
1mm
Map Sum Spectrum
Wt% σ
O 72.9 0.1
C 21.9 0.0
N 5.1 0.0
Cl 0.1 0.0
Powered by Tru-Q®
cps/eV
keV
Cu
C
O
N
Cl

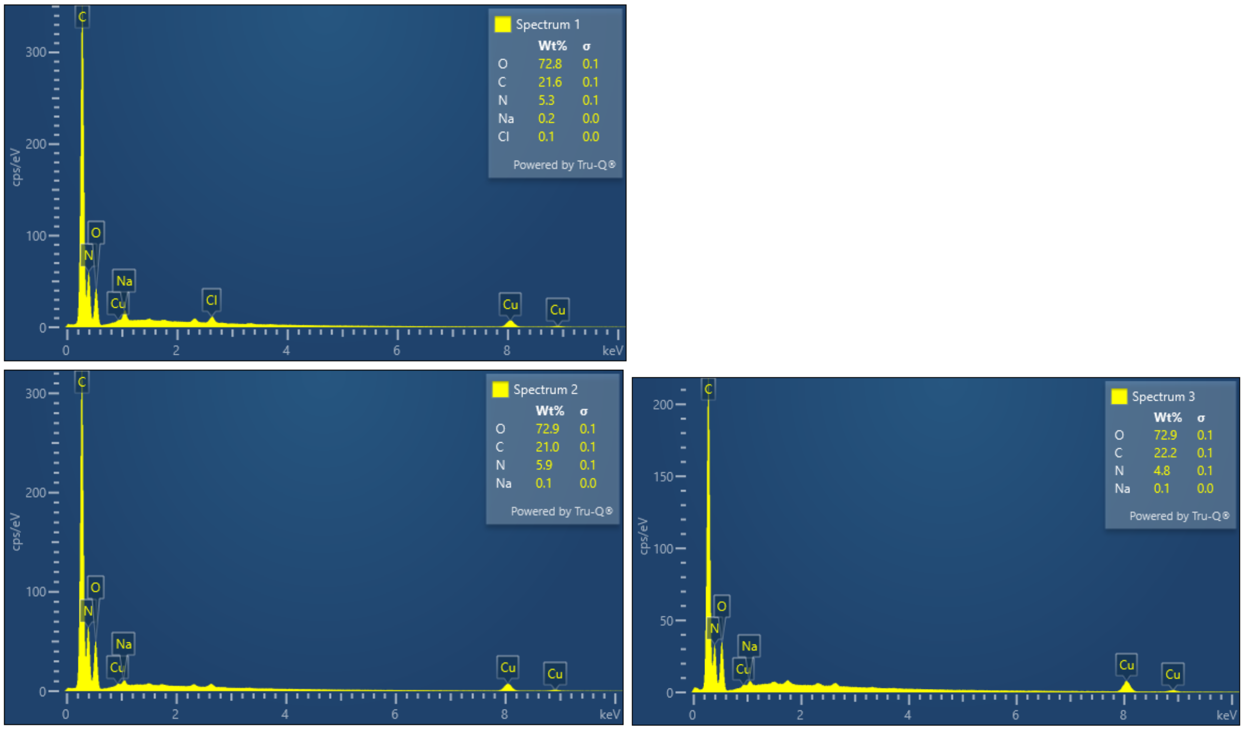

Supplementary Figure 4. (a) Black and white electron image of FN CD locations 1-3. (b) Color coded according to element energy dispersive X-ray spectroscopy (EDS). (c-f) EDS images of sample indicating specific elements. (g) Map sum spectrum weight % of elements at locations 1-3. (h-j) Weight % of elements at locations 1-3.

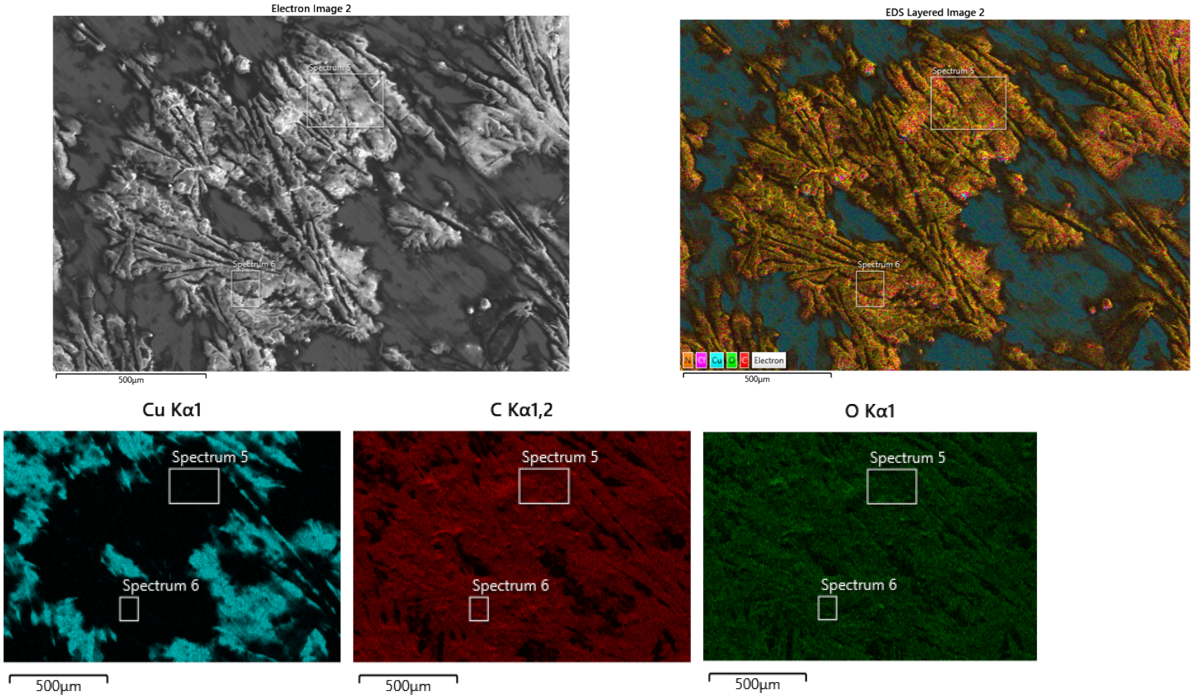

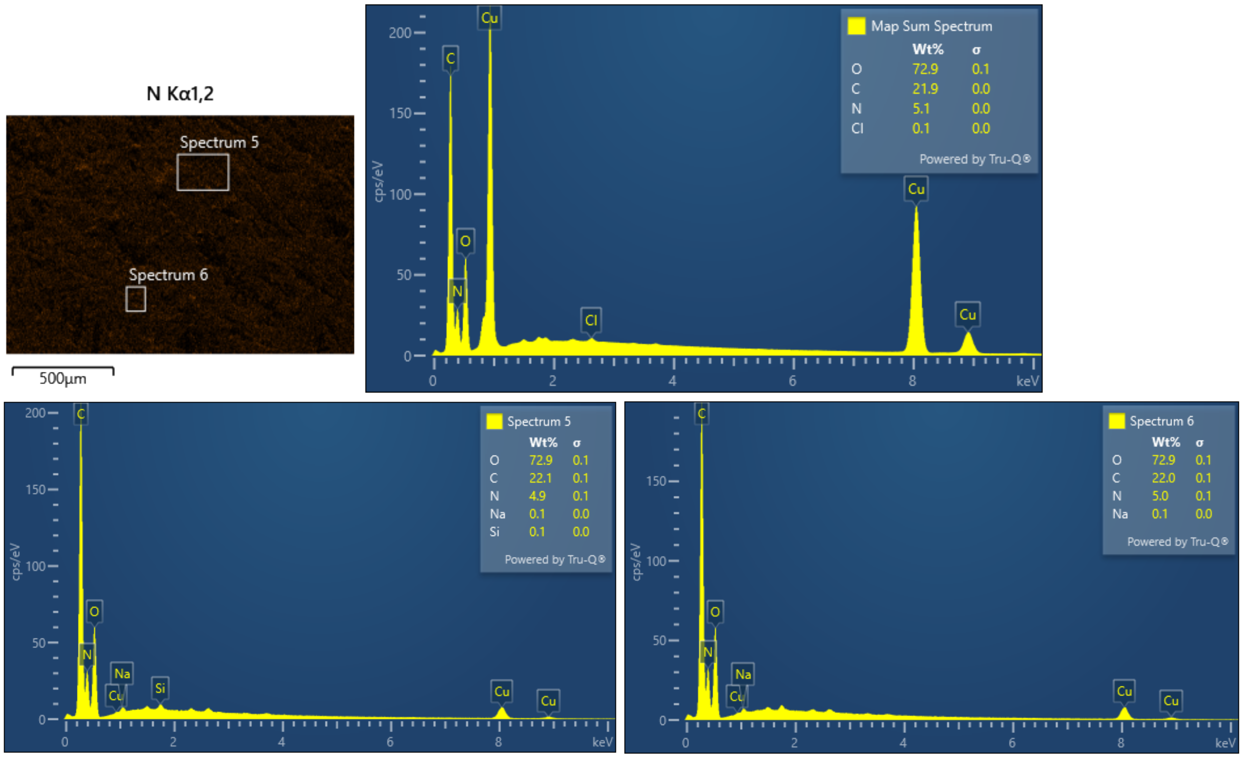

Supplementary Figure 5. (a) Black and white electron image of FN CD locations 5 and 6. (b) Color coded image according to element energy dispersive X-ray spectroscopy (EDS). (c-f) EDS images of sample indicating specific elements. (g) Map sum spectrum weight % of elements at locations 5 and 6. (h and i) Weight % of elements at locations 5 and 6.

Electron Image 3

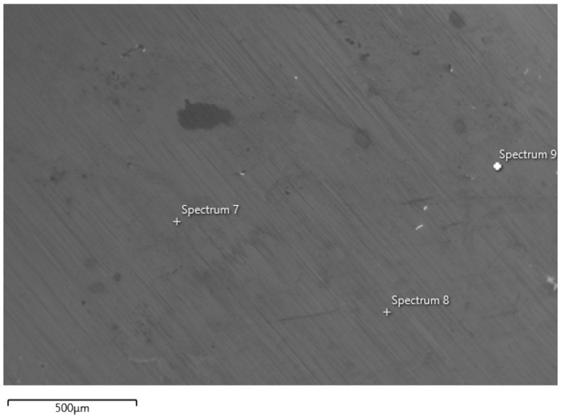

EDS Layered Image 4

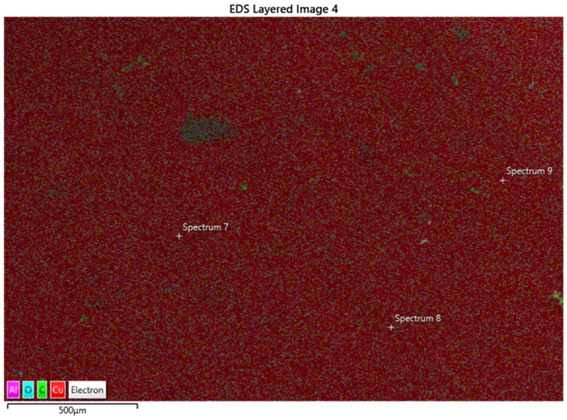

Cu Kα1

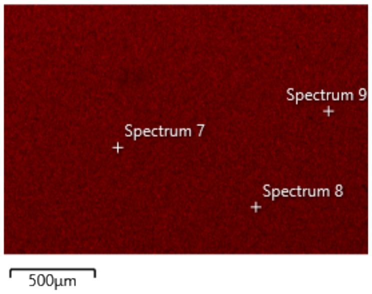

O Kα1

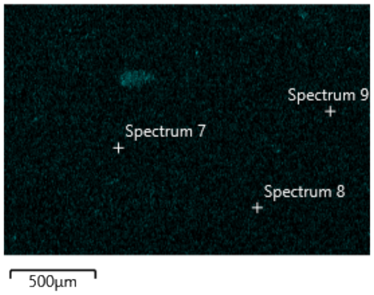

C Kα1,2

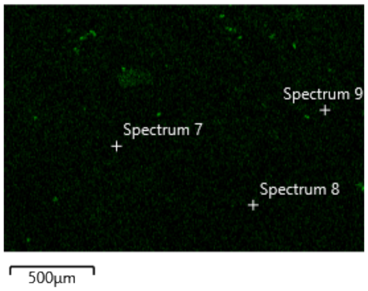

Al Kα1

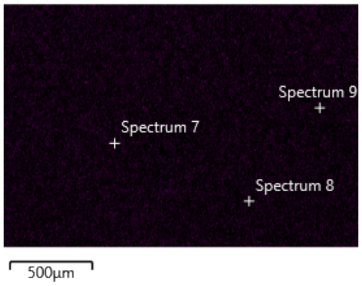

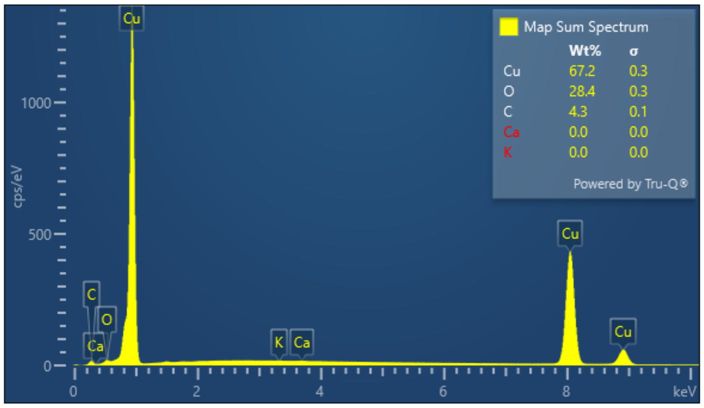

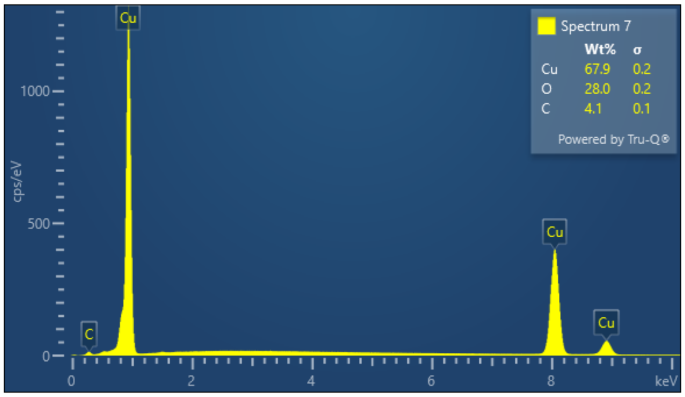

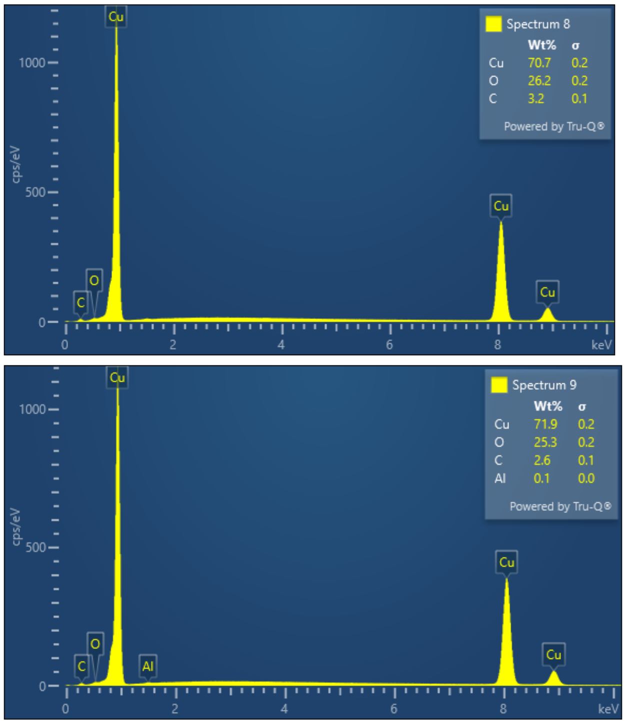

Supplementary Figure 6. (a) Black and white electron image of copper substrate at locations 7-9. (b) Color coded image according to element energy dispersive X-ray spectroscopy (EDS). (c-f) EDS images of substrate indicating specific elements. (g) Map sum spectrum weight % of elements at locations 7-9. (h-j) Weight % of elements at locations 7-9.

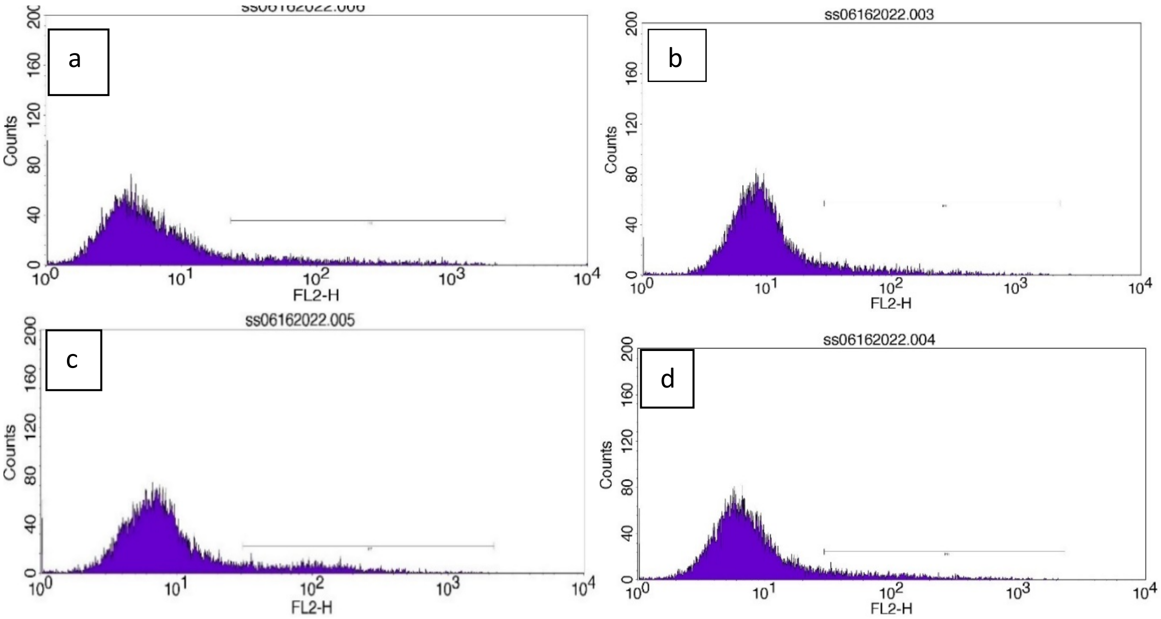

Supplementary Figure 7. (a) Viability control of MDA-321 cell line stained with 10 µL PI . (b,c,d) MDA- 321 cell line stained with 100 µL of FN CDs and 10 µL PI for each sample.
